# Arabidopsis Type III Gγ Protein AGG3 Is a Positive Regulator of Yield and Stress Responses in the Model Monocot *Setaria viridis*

**DOI:** 10.3389/fpls.2018.00109

**Published:** 2018-02-09

**Authors:** Jagdeep Kaur, Swarup Roy Choudhury, Anitha Vijayakumar, Laryssa Hovis, Zach Rhodes, Rob Polzin, Dylan Blumenthal, Sona Pandey

**Affiliations:** Donald Danforth Plant Science Center, St. Louis, MO, United States

**Keywords:** *Setaria viridis*, heterotrimeric G-proteins, AGG3, type III Gγ, yield, stress response

## Abstract

Heterotrimeric G-proteins are key regulators of a multitude of growth and development pathways in eukaryotes. Along with the conserved G-protein components found in all organisms, plants have certain novel variants with unique architecture, which may be involved in the regulation of plant-specific traits. The higher plant-specific type III (or Class C) Gγ protein, which possesses a large C terminal extension, represented by AGG3 in Arabidopsis, is one such variant of canonical Gγ proteins. The type III Gγ proteins are involved in regulation of many agronomically important traits in plants, including seed yield, organ size regulation, abscisic acid (ABA)-dependent signaling and stress responses, and nitrogen use efficiency. However, the extant data, especially in the monocots, present a relatively complex and sometimes contradictory picture of the regulatory role of these proteins. It remains unclear if the positive traits observed in certain naturally occurring populations are due to the presence of specific allelic variants of the proteins or due to the altered expression of the gene itself. To address these possibilities, we have overexpressed the Arabidopsis *AGG3* gene in the model monocot *Setaria viridis* and systematically evaluated its role in conferring agriculturally relevant phenotypes. Our data show that *AtAGG3* is indeed functional in Setaria and suggest that a subset of the traits affected by the type III Gγ proteins are indeed positively correlated with the gene expression level, while others might have more complex, allele specific regulation.

## Introduction

Food security has become an imminent challenge especially with the ever increasing human population on the planet and is now thought to be worse than it was 20 years ago (Alexandratos, 1999). The population of the planet is projected to increase by almost 50% within the next 35 years. With the drastic increase in population, food production also must increase. Without appropriate solutions the need for increased land for crops could irreversibly change terrestrial and aquatic environments (Tilman et al., 2002). Identification of specific genes/targets that confer increased yield potential in non-optimal environment and elucidation of their mode of action is, therefore, central to our future needs. The type III Gγ proteins of the heterotrimeric G-protein complex are fast emerging as one such agronomically important target (Botella, 2012).

Heterotrimeric GTP-binding proteins (G-proteins hereafter) composed of Gα, Gβ, and Gγ subunits mediate signaling in response to a variety of stimuli in all eukaryotes and control critical growth and developmental processes (Urano and Jones, 2014; Stateczny et al., 2016). The G-protein core components and their basic biochemical properties are largely conserved across phyla; however, key differences emerge when comparing plants with metazoan systems. The genomes of most plants encode fewer canonical G-protein subunits, e.g., 1 Gα and 1 Gβ proteins exist in Arabidopsis compared to 23 Gα and 5 Gβ proteins in humans (Hackenberg et al., 2017). Intriguingly, plant genomes also encode certain divergent G-protein components, which may be involved in the control of plant-specific functions. One such plant-specific component is exemplified by the novel type III Gγ proteins, which are emerging as a major target for plant breeding (Chakravorty et al., 2011; Roy Choudhury et al., 2011).

The plant Gγ proteins are classified into three different groups based on their C-terminal region. The type I are the canonical Gγ proteins found in all organisms. These are 100–120 aa proteins, represented in Arabidopsis by AGG1 and AGG2. The type II Gγ proteins are very similar to the type I Gγ proteins but they lack the signature C-terminal prenylation motif present in canonical Gγ (Roy Choudhury et al., 2011; Thung et al., 2012). Both type I and type II Gγ proteins have been shown to be involved in plant-microbe interaction and various hormone signaling pathways (Trusov et al., 2006, 2007, 2008, 2009; Delgado-Cerezo et al., 2012; Yadav et al., 2012; Roy Choudhury and Pandey, 2013).

The type III Gγ proteins, represented by Arabidopsis AGG3, rice DEP1, GS3 and GGC2, wheat TaDEP1, barley HvDEP1 and soybean GmGγ8, GmGγ9, and GmGγ10 are at least twice as large as the type I or type II proteins and have a modular architecture (Roy Choudhury et al., 2011; Botella, 2012; Trusov et al., 2012). The N-terminal region of these proteins is similar in size and sequence to the type I and II Gγ proteins and is connected with the C-terminal region with a putative transmembrane domain. The C-terminal region is extremely rich in amino acid Cysteine (Cys), which can account for up to 38% of total amino acids in this region (Roy Choudhury et al., 2011). Interestingly, there is an expansion of the C-terminal region in plants that have more than one homolog of type III Gγ protein. For example, the three rice proteins possess 100, 200, and 300 amino acids in their C-terminal region, while the N-terminal region is fairly conserved. Similar expansion of the C-terminal region is seen in the soybean type III Gγ proteins. This unique Cys-rich region has predicted segments showing some similarity to tumor necrosis/nerve growth factor receptor (TNFR/NGFR) and multiple repeats of the von Willebrand factor type C modules and a Sprouty domain, which are thought to be involved in large protein complex formation (Roy Choudhury et al., 2011; Botella, 2012; Trusov et al., 2012; Wolfenstetter et al., 2014).

Type III Gγ proteins regulate two of the most critical plant processes; seed yield and stress responses. The *AGG3* gene of Arabidopsis was discovered as the missing Gγ protein that could explain a subset of Gβ mutant phenotypes, especially those related to ABA-responses, unaccounted for by the previously identified *AGG1* and *AGG2* genes (Chakravorty et al., 2011; Wolfenstetter et al., 2014). Additionally, an independent study in Arabidopsis identified *AGG3* by map-based cloning as an organ size regulator, as loss-of-function of *AGG3* resulted in smaller leaves and flowers (Li et al., 2012b). Surprisingly, a survey of rice literature revealed that the homologs of this gene were already characterized, although not as Gγ protein. The rice proteins, named GS3 (grain size 3) and DEP1 (dense and erect panicle 1), were identified as major quantitative trait loci (QTL) for panicle density, seed size and seed number (Fan et al., 2006; Huang et al., 2009; Mao et al., 2010; Li et al., 2012a; Kunihiro et al., 2013).

Several studies in the past years have revealed a relatively complex picture of the type III Gγ regulated processes in plants. Overall, the situation seems to be clearer in dicots such as Arabidopsis and Camelina, where AGG3 protein has been shown to be a positive regulator of stress response and organ size. Overexpression of *AGG3* in both Arabidopsis and Camelina results in larger plants, bigger seeds and better stress tolerance, while the knockout mutants of *AGG3* in Arabidopsis have an opposite phenotype suggesting a direct, positive correlation between the protein level and the observed phenotypes (Chakravorty et al., 2011; Li et al., 2012b; Roy Choudhury et al., 2014; Alvarez et al., 2015). However in monocots, especially in rice where the gene has been studied extensively at the genetic level, extant data present a complex scenario.

The *GS3* gene, as the name suggests, regulates grain size in rice and plants with different allelic variants of this gene produce differently sized grains (Mao et al., 2010). Varieties containing naturally occurring mutations resulting in the potential loss-of-function alleles produce extremely long grains, whereas a variant causing deletion of the C-terminal region but leaving the most of the Gγ-like domain intact, results in plants producing extremely short grains. Additional variants which produce normal, short or long grains, depending on the location of the mutation are also reported (Fan et al., 2006, 2009; Mao et al., 2010; Botella, 2012). Overall, it has been concluded that the GS3 locus is a negative regulator of grain size, which incidentally is opposite of the role of *AGG3* gene in Arabidopsis and Camelina.

Different allelic variants of the *DEP1* locus, which was initially identified as a major QTL for dense and erect panicles, also confer distinct phenotypes (Huang et al., 2009). For examples plants possessing *dep1* or *qPE9-1* alleles, which code for almost identical proteins with only one amino acid difference in their lengths have different phenotypes. *dep1*, which is a gain of function mutation, leads to increased panicle branching and improved grain yield whereas *qPE9-1*, which is a loss-of-function mutation, exhibits no change in branching and causes reduced yield (Huang et al., 2009; Zhou et al., 2009; Yi et al., 2011). RNAi-mediated suppression of *DEP1* locus in *dep1* allelic background resulted in curved panicles and fewer grains, whereas expression of a *DEP1* promoter-driven expression of *dep1* allele resulted in erect panicles and increased yield. Furthermore, a constitutive promoter-driven expression of the *dep1* resulted in dwarf plants with erect panicles, whereas similar expression of *DEP1* allele had no phenotypic effect. Finally, a recent CRISPR/CaS9 based editing of *DEP1* gene resulted in erect panicles, similar to what was reported with the *dep1* allele, but the plants were also dwarfed and the grain size was not affected, which is not what was seen with the naturally occurring *dep1* mutation (Li et al., 2016; Xu et al., 2016; Zhao et al., 2016). In wheat, an RNAi-mediated downregulation of *TaDEP1* led to longer and less compact spikes, whereas a similar loss-of-function mutation in barley *HvDEP1* resulted in dwarf plants, with compact, shorter spikes and improved seed yield (Wendt et al., 2016). Recently *DEP1* has also been identified as a major QTL for nitrogen use efficiency (NEU) in rice (Sun et al., 2014; Wendt et al., 2016; Xu et al., 2016). Finally, a 12 year field study in barley concluded that the effect of *DEP1* locus is highly dependent on the environmental conditions and may result in significantly higher or lower yields, compared to the wild type control plants (Wendt et al., 2016).

Based on these studies, the overall consensus is that the type III *Gγ* genes are critical regulators of important agronomic traits, and have been subjected to artificial selection all through domestication. However, there seem to be a huge effect of the genetic background as well as specific environmental conditions that determine the eventual yield and stress responses of the plants possessing specific alleles. To gain a better understanding of the role of type III Gγ proteins, we have overexpressed a monocot codon optimized *AGG3* gene in *Setaria viridis* (green foxtail), a member of the Panicoideae subfamily of grasses which also include food crops like maize and sorghum. The availability of a sequenced genome, efficient transformation system and rapid life cycle with ample seed production makes it an ideal model system for the study of physiological, developmental and yield traits of important grain crops (Acharya et al., 2017). The data presented in this manuscript confirm that the *AGG3* gene is functional in Setaria and while some of the traits are indeed positively regulated by constitutive overexpression of *AGG3* gene, others might have a more complex regulation, dependent on the presence of specific alleles, genetic background or environmental conditions.

## Materials and Methods

### Construction of Plant Expression Vectors and *S. viridis* Plant Transformation

A 759 bp *AGG3* (*At5g20635*) gene (Supplementary Figure S1) was chemically synthesized (GeneScript Incorporated, Piscataway, NJ, United States) using the monocot-preferred codons. Employing Gateway^®^ (GW) strategy, the full-length *AGG3* gene was cloned into *pCR8/GW/TOPO* vector (Invitrogen, Waltham, MA, United States) using manufacturer’s instructions. The resulting *pCR8/GW/AGG3* entry clone after sequence confirmation was recombined into *pANIC10A* expression vector (Mann et al., 2012) using LR clonase enzyme (Invitrogen, Waltham, MA, United States) which allowed constitutive expression of *AGG3* driven by *ZmUbi1* (*Zea mays* ubiquitin 1) promoter. The sequence confirmed *pANIC10A::AGG3* and *pANIC10A* (empty vector, EV, hereafter) constructs were transformed into *Agrobacterium tumefaciens* strain AGL1 using standard protocol. Transgenic *S. viridis* (A10.1) plants expressing these two constructs were generated by the plant transformation facility at Boyce Thompson Institute, Ithaca, NY (Van Eck and Swartwood, 2015). The transformed *S. viridis* T_0_ events were genotyped for the presence of selectable marker gene *hph* using primers listed in Supplementary Table S1. T_0_ events positive for *hph* gene were grown to maturity and T_1_ seeds were shipped to the Danforth Center for further characterization.

### Characterization of Transgenic *S. viridis* Plants

Seeds were propagated by growing in metro mix 360 potting mix (Hummert International, Earth City, MO, United States), in a growth chamber which was maintained at 31°C day/ 22°C night temperature, with a relative humidity 50–60% at a 12 h day/12 h night photoperiod. Plants were watered once a day and fertilized twice a week.

One hundred mg of leaf tissue from the T_1_ families of transgenic plants (*AGG3-OE* and *EV*) was used for DNA isolation following a CTAB method. DNA was quantified on a Nanodrop 2000c (Thermo Fisher Scientific, Austin, TX, United States) and used for genotyping, Southern blot analysis and TaqMan assays. Plants carrying the *AGG3* transgene were identified by genomic PCR. To determine the insert integration pattern a DIG (Digoxigenin)-labeled Southern hybridization protocol was used as described in https://docs.wixstatic.com/ugd/45ed6d_bbc4921f988e4aa7afd873237555a42a.pdf. Briefly, 10 μg of DNA was digested with *Mfe*I, run on a 1.0% agarose gel and transferred to a positively charged nitrocellulose membrane. The membrane was UV-crosslinked and prepared for hybridization (pre-hybridization) using the DIG Easy Hyb (Roche, Indianapolis, IN, United States) solution. The hybridization probe complimentary to the *hph* gene was prepared using DIG-labeled dNTPs and *pANIC10A* plasmid DNA as template. The membrane was hybridized overnight followed by washing with low (2X SSC, 0.1% SDS) and high (0.5 X SSC, 0.1% SDS) stringency washes and blocked with 1X blocking buffer (1X maleic acid buffer, Blocking Reagent- Roche). Afterward the membrane was treated with anti-DIG AP Fab Fragments (Roche) prepared in blocking buffer. The membrane was washed three times with 1X washing buffer (1X maleic acid buffer, Tween 20). Detection was done using the CDP-Star reagent (Thermo Fisher Scientific, St. Peters, MO, United States) for 5 min.

To identify the homozygous families from single insert transgenic *S. viridis AGG3-OE* and *EV* lines, a TaqMan assay was performed using https://docs.wixstatic.com/ugd/45ed6d_4ce1ccdacf3243c794ad1f9f9f19b8b3.pdf. Briefly, the multiplex reaction (10 μL total) contained 5 μL of genotype master mix (Thermo Fisher Scientific, St. Peters, MO, United States), 0.3 μL of nuclease-free water, 0.9 μL of each of *hph* (marker gene) and *SvPCKR* (*S. viridis* phosphoenolpyruvate carboxykinase gene, internal control) forward and reverse primers, 0.025 *hph* (5′ FAM 3′ QSY) and *SvPCKR* (5′ VIC 3′ TAMRA) dually labeled probes (see sequence information of primer and probes in Supplementary Table S1) and 1 μL of genomic DNA (1 μg equivalent). Each sample including wild type A10.1 and non-template controls (NTC) was run in triplicate. The data were imported into CopyCaller Software (Thermo Fisher Scientific, Carlsbad, CA, United States) and analyzed for CNV, without the use of a calibrator. The software generated a graphical output of the copies of the gene of interest that were present in the genome of each individual.

### RNA Isolation, DNase Treatment, cDNA Synthesis, and qRT-PCR Analysis

Total RNA from *EV* and *AGG3-OE* transgenic plants was extracted from 2 weeks old seedlings using TRIzol reagent and was digested with RNase-free DNaseI (Ambion^®^, Thermo Fisher Scientific). The quantity and the quality of the RNA were assessed with nanodrop spectrophotometer. Total RNA (500 ng) was reverse transcribed into cDNA using first-strand cDNA synthesis kit (Invitrogen, Carlsbad, CA, United States) and used for quantitative real-time PCR (qPCR) using SYBR mix (Invitrogen). qRT-PCR was conducted in 10 μL reaction mix in three biological replicates. Similar setup was used to check the expression levels of a subset of nitrate transporter and signaling genes. Differences in transcript level were calculated using the ^ΔΔ^CT method (Bustin et al., 2009). Data represent the means and standard errors (SE) of three biological replicates. The gene specific primers and the reference gene used in the qPCR reactions are listed in Supplementary Table S1.

### Germination and Early Seedling Growth Assays

Sterilized Setaria seeds were plated on 0.5 X MS media (Caisson labs, Smithfield, UT, United States) with 0.4% phytagel. Seeds were stratified at 4°C in the darkness for 2 days followed by transfer to the growth chamber maintained at a 12 h (31°C)/ 12 h (22°C) light/dark cycle (Acharya et al., 2017). Germination was defined as protrusion of the radicle from seeds and quantified as the percentage of total seeds at 5 days after plating. To quantify the seed germination in the presence of ABA or glucose, sterilized seeds were plated directly on 0.5 X MS media containing 0.5 μM ABA or 3% glucose. Three biological replicates of each experiment were performed with 24 seeds per genotype per treatment per replicate and data were averaged. Significant differences were analyzed using the Student’s *t*-test.

To examine early seedling growth, Setaria seeds sown on 0.5 X MS media plates were stratified at 4°C in the dark for 2 days followed by transfer to the growth chamber with 12 h (31°C)/ 12 h (22°C) light/dark cycle for another 2 days. To evaluate the effect of ABA, glucose or salt (NaCl) on post-germination growth, germinated seeds were transferred on plates containing 2 μM ABA, 3% glucose or 100 mM NaCl (Acharya et al., 2017). Seedlings (20–24 seedlings per genotype per treatment) were grown vertically in the 12 h dark/12 h light cycle and coleoptile and root lengths were measured after 3 days of growth. Germinated seeds transferred to control plates and grown under identical conditions were used as control. To determine the effect of nitrogen or phosphorus limiting conditions, seeds were plated on nitrogen or phosphate deficient 0.5 X MS media (Caisson Labs), and coleoptile and root lengths were measured after 7 days of growth. All experiments were repeated three times and data were averaged.

### Adult Plant Growth and Development Assays and Stress Treatment

Plants were grown by sowing the seeds directly into 10 cm pots containing metro mix 360 potting mix in the environmentally controlled greenhouse maintained at 50–60% relative humidity, 31°C/22°C day/night temperature and 12 h of day length (250 μmol m^-2^ s^-1^) to maturity (Acharya et al., 2017). The phenotypic parameters such as plant height, leaf number, days to heading, size and the number of the panicles were measured weekly. Plants were also grown under low water (50% of the water compared to the well-watered control), nitrogen limiting (no exogenous N_2_ added during fertilization versus 15 mM added in control set), and a combination of low water, N_2_ limiting conditions. After 8 weeks of growth, plants were allowed to dry and bagged to avoid seed loss. Seeds were collected from completely dried plants. Each experiment contained 12 plants per genotype and the experiment was repeated three times, independently. Data were averaged and are presented as the mean of three biological replicates. Significant differences between EV and transgenic plants’ phenotypes was evaluated using Student’s *t*-test.

### Statistical Analysis

All experiments were repeated at least three times independently and data were averaged. Means, standard deviation (seed germination assays) and standard errors (for root length and number, coleoptile length, leaf number, plant height, panicle number and size, seed weight and qRT-PCR) for measurements were calculated. Statistical significance of results was calculated using Student’s *t-*test with a *P*-value threshold of ≤0.05 (^∗^), ≤0.01 (^∗∗^) or ≤0.001(^∗∗∗^).

## Results

### Generation of Monocot Codon Optimized *AGG3* Overexpressing (*AGG3-OE*) *S. viridis* Plants

The homologs of type III *Gγ* genes are present in all higher plants. Using Arabidopsis *AGG3* and rice *DEP1* and *GS3* as query sequences we identified three type III *Gγ* genes *Sevir.6G177400.1, Sevir.2G229300.1* and *Sevir.9G375000.1* referred as *SvGG3a, SvGG3b* and *SvGG3c*, respectively, in the *Setaria viridis* genome (Supplementary Table S1). These genes share ∼25%, 31% and 29% identity, respectively, with Arabidopsis *AGG3*; ∼24%, 57% and 22% identity, respectively, with the rice *DEP1* and ∼21%, 20% and 46% identity, respectively, with the rice *GS3* at the amino acid level. The homology mostly exists within the Gγ-like domain of the protein sequences (Supplementary Table S2) and is typical of sequence homologies found within the Gγ proteins. Each one of these Setaria Gγ proteins might be involved in the regulation of one or more developmental, yield-related or stress-response pathways. Because our goal was to evaluate the extent to which the response regulation is dependent on the prototypical type III Gγ protein’s expression level, and not on the presence of a specific variant or allele in the genome, we decided to overexpress a monocot codon-optimized version of the Arabidopsis *AGG3* gene and assess its effect on plant growth, development, yield, and stress response in Setaria.

**FIGURE 1:**
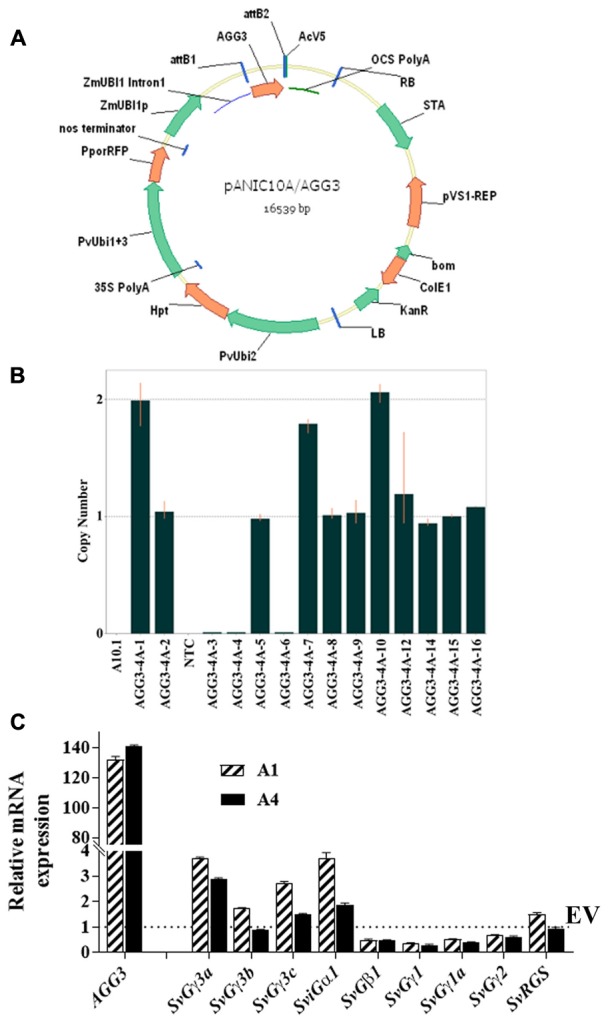
Generation of Setaria *AGG3* overexpression lines. **(A)** Map of the expression vector used for cloning a monocot codon optimized Arabidopsis *AGG3* gene. **(B)** Identification of the homozygous families from single insert AGG3-4A transgenic plants in T1 generation using Taqman assay **(C)** Expression analysis of *AGG3* transgene and endogenous Setaria G-protein genes by qRT-PCR analysis. Transcript levels of indicated genes were determined in *EV* and *AGG3-OE* (A1 and A4) lines using cDNA isolated from 10-day-old *S. viridis* seedlings. The expression values of EV, A1 and A4 were normalized with Setaria ubiquitin gene. Dotted line represents the expression level of *SvGγ3a, SvGγ3b, SvGγ3c, SviGα1, SvGβ1, SvGγ1, SvGγ1a, SvGγ2, SvRGS* in the EV (assigned as 1). The values are presented as the mean ± SE of three biological replicates and represented as fold changes. EV, Setaria plants overexpressing the empty vector; A1, A4 are the two independent transgenic lines for *AGG3* overexpression (*AGG3-OE).*

For strong constitutive expression, the monocot codon-optimized *AGG3* (Supplementary Figure S1) was driven by maize *ZmUbi1* promoter and intron, flanked by octapine synthase polyadenylation signal in *pANIC10A::AGG3* (**Figure 1A**). We obtained eight and two independent T_0_ events belonging to *pANIC10A::AGG3* and *EV* constructs, respectively. Between 13 and 16 T_1_ families/event were tested for the presence/absence of the plant selectable marker *hph* gene and the data were subjected to goodness of fit for single locus Mendelian segregation of 3:1 using chi-squared analysis. As reported in **Table 1**, 6/8 events (AGG3-1A, AGG3-2A, AGG3-3A, AGG3-4A, AGG3-5A, and AGG3-6B) for *pANIC10A::AGG3* and 2/2 events (EV-2A and EV-3A) for *EV* construct showed single locus inheritance. T_1_ events AGG3-1B and AGG3-6A did not conform to 3:1 segregation (**Table 1**).

**Table 1:** Genetic and molecular characterization of transgenic T_1_ *S. viridis* lines.

Line	PCR segregation		Chi-square (χ^2^)	Insert number^b^	Zygosity^c^			Chi-square (χ^2^)
	*hph+*	*hph-*	Value (3:l)^a^		Homo	Hetero	Null	Value (l:2:l)^a^
*pANIC10A::AGG3*								
AGG3-1A	14	1	2.69	2		Not Tested		
AGG3-1B	16	0	5.33^∗^	2		Not Tested		
AGG3-2A	14	2	1.33	2		Not Tested		
AGG3-3A	9	5	0.86	1	4	5	3	0.50
AGG3-4A	11	3	0.94	1	3	8	3	0.28
AGG3-5A	14	2	1.33	3		Not Tested		
AGG3-6A	16	0	5.33^∗^	3		Not Tested		
AGG3-6B	15	1	3.00	3		Not Tested		
*pANIClOA (empty vector, EV)*								
EV-2A	12	2	0.86	1	3	9	3	0.60
EV-3C	12	4	0.00	1	6	5	3	2.42

*^*a*^At 1 and 2 degree of freedom and P = 0.05, χ^*2*^ critical values are 3.84 and 5.99, respectively. ^*b*^Based on Southern hybridization data on pooled Ti families. ^*c*^Based on TaqMan assay, Homo (homozygous), Hetero (Heterozygous) and Null transgenic lines were identified. ^∗^Significant at P = 0.05.*

All T_1_ events showing 3:1 segregation were tested for stable integration of the expression cassette using Southern hybridization using a DIG-labeled *hph* probe. Events AGG3-1A and AGG3-2A showed the presence of two copies, AGG3-3A and AGG3-4A carried a single copy while AGG3-5A and AGG3-6A showed three copies of the insert. Both the *EV* events EV-2A and EV-3A showed the presence of single insert. From the distinct banding pattern, all of these lines seemed to be independent events (Supplementary Figure S2). We also used a TaqMan assay to identify homozygous families from single copy events. Thus, 4, 3, 3 and 6 homozygous lines each from AGG3-3A, AGG3-4A, EV-2A and EV-3A, respectively, were identified (**Table 1**). As expected, these single copy events conformed to 1:2:1 segregation pattern (**Table 1**). **Figure 1B** shows representative data for AGG3-4A event, where families numbered AGG3-4A-1, -7, and -10 were homozygous with 2 copies, while families -2, -5, -8, -9, -12, -14, -15 and -16 were heterozygous. PCR null families -3, -4 and -6 were nulls. No amplification was observed in wild type A10.1 and NTC control as expected (**Figure 1B**).

The T_1_ seeds from *pANIC10A::AGG3* and *EV* events were self-fertilized to obtain T_2_ seeds. Seeds from each of the T_2_ family were collected individually. Sixteen T_2_ families from each event were progeny tested for *hph* gene (**Table 2**). Based on these data, AGG3-1A-10, AGG3-1B-15, AGG3-1B-16, AGG3-2A-2, AGG3-2A-7, AGG3-3A-9, AGG3-4A-7, AGG3-5A-2, AGG3-6A-4, AGG3-6A-6, AGG3-6B-12, EV-2A-5 and EV-3C-2 families were all PCR positives and considered homozygous, while AGG3-5A-1 and AGG3-6B-13 were segregating (**Table 2**). All the events were advanced to T_3_ generation by self-fertilization. Under normal growth and development conditions no phenotypic differences were observed between different T3 lines (Supplementary Figure S3). Two independent, homozygous lines, designated as A1 (AGG3-1B-15-1) and A4 (AGG3-4A-7-4), and the EV containing line EV-2A-5-1 (EV) were selected for further molecular and phenotypic characterization (**Table 2**).

**Table 2:** Genetic and molecular characterization of transgenic T_2_ and T_3_ *S. viridis* lines.

T_2_ generation				T_3_ generation
Line	PCR segregation^a^		Zygosity status	Family selected for further characterization and their designation^b^
	*hph*+	*hph -*		
*pANIC10A::AGG3*				
AGG3-1A-10	16	0	Homo	/
AGG3-1B-15	16	0	Homo	AGG3-1B-15-1 (Al)
AGG3-1B-16	16	0	Homo	/
AGG3-2A-2	16	0	Homo	AGG3-2A-2-3 (A2)
AGG3-2A-7	16	0	Homo	/
AGG3-3A-9	16	0	Homo	AGG3-3A-9-2 (A3)
AGG3-4A-7	16	0	Homo	AGG3-4A-7-4 (A4)
AGG3-5A-1	5	11	Segregating	/
AGG3-5A-2	16	0	Homo	AGG3-5A-2-2 (A5)
AGG3-6A-4	16	0	Homo	/
AGG3-6A-6	16	0	Homo	AGG3-6A-6-1 (A6)
AGG3-6B-12	16	0	Homo	AGG3-6B-12-4(A7)
AGG3-6B-13	13	3	Segregating	
*pANIClOA (empty vector, EV)*				
EV-2A-5	16	0	Homo	EV-2A-5-1 (EV)
EV-3C-2	16	0	Homo	EV-3C-2-1 (EV-2)

*^*a*^Absence of hph negative plants indicate homozygosity (Homo) status of parent line. ^*b*^Families were phenotyped.*

### Expression Analysis of *AGG3* and Native G-Protein Genes in Transgenic *S. viridis* Plants

To confirm the higher expression level of the introduced transgene, the transcript level of *AGG3* in Setaria was quantified using qPCR. The analysis showed more than 100 fold increase in the level of *AGG3* transcript in A1 and A4 lines compared to the ubiquitin gene, which was used as control. No *AGG3* transcript was detected in the EV containing plants. Because G-proteins typically work as a protein complex, we also determined the expression levels of other native G-protein genes of Setaria (gene names and accession numbers listed in Supplementary Table S1) upon *AGG3* overexpression (**Figure 1C**). The transcript levels of the Setaria G-protein complex genes were not significantly and consistently different between EV control versus A1 and A4 plants. These data confirm that a higher expression of *AGG3* does not significantly affect the expression level of other members of the G-protein complex. This also ascertains that any differences observed in the overall traits of the transgenic plants is indeed due to the overexpression of *AGG3* and not due to alteration in the level of other proteins of the complex.

### Effect of *AGG3* Overexpression on Early Plant Development

Constitutive overexpression of the *AGG3* has an overall positive effect on the growth and stress responses in Arabidopsis and Camelina, the two species where it has been evaluated. The effects of *AGG3* overexpression in Camelina are obvious from the early seedling stage as the transgenic plants are bigger and more robust (Roy Choudhury et al., 2014). However, in Setaria, the germination and early seedling growth of EV containing plants was indistinguishable from the A1 and A4 plants when grown on synthetic media plates, under control conditions. We then evaluated the effect of different stresses on early development as it has been shown that higher expression of *AGG3* results in hyposensitivity to exogenous ABA during seed germination and early seedling growth in Arabidopsis, and the knockout mutants of *AGG3* gene are hypersensitive to ABA (Chakravorty et al., 2011). Similarly, transgenic Camelina plants overexpressing *AGG3* exhibit reduced sensitivity to ABA as well as other abiotic stresses such as exogenous sucrose and NaCl during germination and early seedling growth (Roy Choudhury et al., 2014; Alvarez et al., 2015). Incidentally, in monocot plants, the functional role of type III *Gγ* genes related to stress response has been not investigated in sufficient detail. There is one study in rice where *qPE9-1* (a mutation in *DEP1* locus) has been shown to negatively regulate ABA responses during seed germination and post-germination root growth (Zhang et al., 2015) suggesting that the stress-related regulatory role of AGG3 and its homologs might be conserved among dicots and monocots.

**FIGURE 2:**
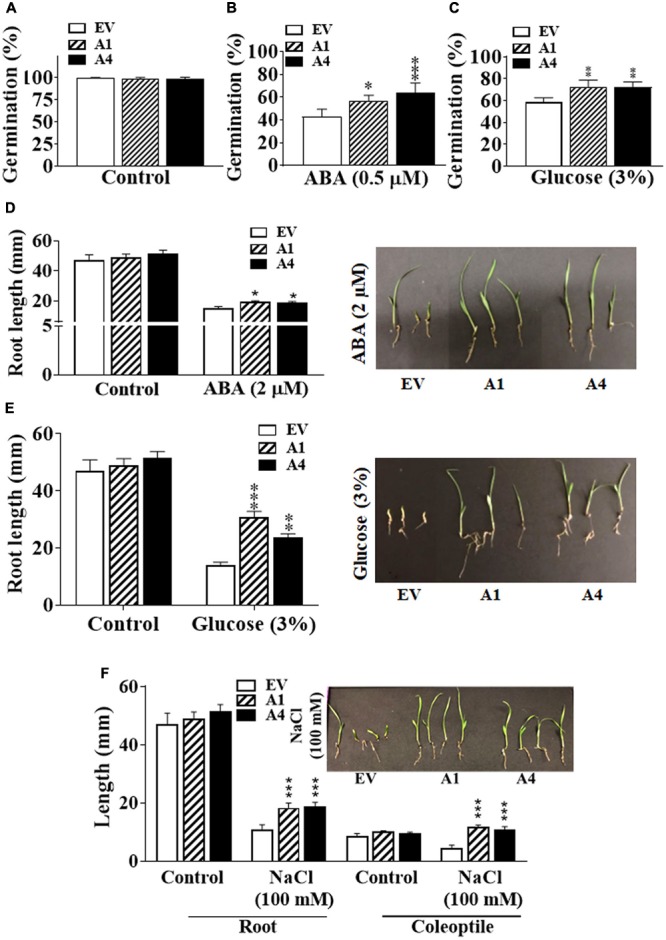
Effect of different abiotic stresses on early development of Setaria *AGG3OE* plants. **(A)** Percentage of seed germination in EV, A1, and A4 transgenic seeds under control conditions. **(B)** Percentage of seed germination in EV, A1 and A4 transgenic seeds in the presence of 0.5 X MS media containing 0.5 μM ABA or **(C)** 3% glucose. For these experiments, 24 seeds per genotype, per treatment were used. Seeds were sterilized and stratified at 4°C for 2 days. Seed germination was calculated as the percentage of total seeds that germinated after 5 days post-stratification. The data represent mean values (±SE) of three biological replicates. **(D)** Post-germination seedling growth of EV and A1 and A4 transgenic plants in the presence of 2 μM ABA, **(E)** 3% glucose or **(F)** 100 mM NaCl. Seeds were germinated on 0.5 X MS agar for 2 days followed by their transfer to the media containing different additives and grown for another 3 days. Root and coleoptile lengths were measured. The graphs represent the mean values (±SE) from sixty seedlings. The corresponding representative pictures are also shown. Asterisks represent *P*-values ≤ 0.05 (^∗^), ≤0.01 (^∗∗^), or ≤0.001 (^∗∗∗^) as calculated using Students *t-*test.

Seeds of both A1 and A4 transgenic plants germinated similar to the EV seeds in the ABA-free medium, indicating that there is no change in their sensitivity to endogenous ABA (**Figure 2A**). However, in the presence of 0.5 μM exogenous ABA, the germination of A1 and A4 seeds was considerably improved compared to the EV seeds, and a clear ABA hyposensitivity was observed. Five days after plating, approximately 55–65% of A1 and A4 seeds had germinated, respectively, compared with 40% germination observed in EV seeds on ABA (0.5 μM) containing media (**Figure 2B**).

Because *AGG3* gene is also known to regulate sugar sensitivity and both ABA and glucose signaling pathways are intricately linked (Rook et al., 2006; Seki et al., 2007; Hey et al., 2010; Vishwakarma et al., 2017), we investigated whether the overexpression of *AGG3* resulted in altered responsiveness to glucose. Similar to what was observed for ABA, the A1 and A4 seeds showed better germination compared with the EV seeds in the presence of exogenous glucose. After 5 days of growth on 3% glucose containing media, ∼70% germination was seen in the A1 and A4 seeds, compared with ∼55% germination in EV seeds (**Figure 2C**).

Inhibition of primary root length in the early seedling stage is one of the important phenotypic effects of ABA or glucose-mediated responses (Fedoroff, 2002; Rook et al., 2006; Hey et al., 2010). We compared the effect of exogenous ABA and glucose on primary root length of the EV, A1 and A4 seedlings. Under control conditions, the primary root lengths of all plants were comparable. However, similar to the ABA-mediated inhibition of seed germination, the A1 and A4 seedlings showed less sensitivity to ABA for primary root length inhibition. In the presence of 2 μM exogenous ABA, the primary root length of EV containing seeds was inhibited by ∼57%, compared to ∼43% and ∼36% inhibition observed in A1 and A4 seedlings, respectively (**Figure 2D**). Similar results were obtained in the presence of 3% glucose where the primary roots of the A1 and A4 seedlings was significantly bigger compared to the EV seedlings. Almost 60% reduction in root length was seen for the EV seeds compared to ∼35% and 40% reduction seen in A1 and A4 seedlings, respectively (**Figure 2E**) in the presence of glucose.

We have previously shown that overexpression of *AGG3* gene also enhances salt tolerance in transgenic Camelina (Roy Choudhury et al., 2014). To evaluate the salt tolerance of *AGG3*-overexpressing Setaria plants, seeds were first germinated on 0.5 X MS media and after 2 days of growth, transferred to 0.5 X MS media supplemented with 100 mM NaCl. In the presence of NaCl, A1 and A4 seedlings grew larger than those of the EV seedlings, exhibiting significantly increased primary root length (1.7 times bigger that EV roots) and coleoptile length (2.5 times bigger than EV coleoptiles), exhibiting a hyposensitive response to salt stress (**Figure 2F**). Taken together, these data suggest a general improvement of stress tolerance in the *AGG3*-overexpressing transgenic Setaria plants during germination and at the early seedling stage.

**FIGURE 3:**
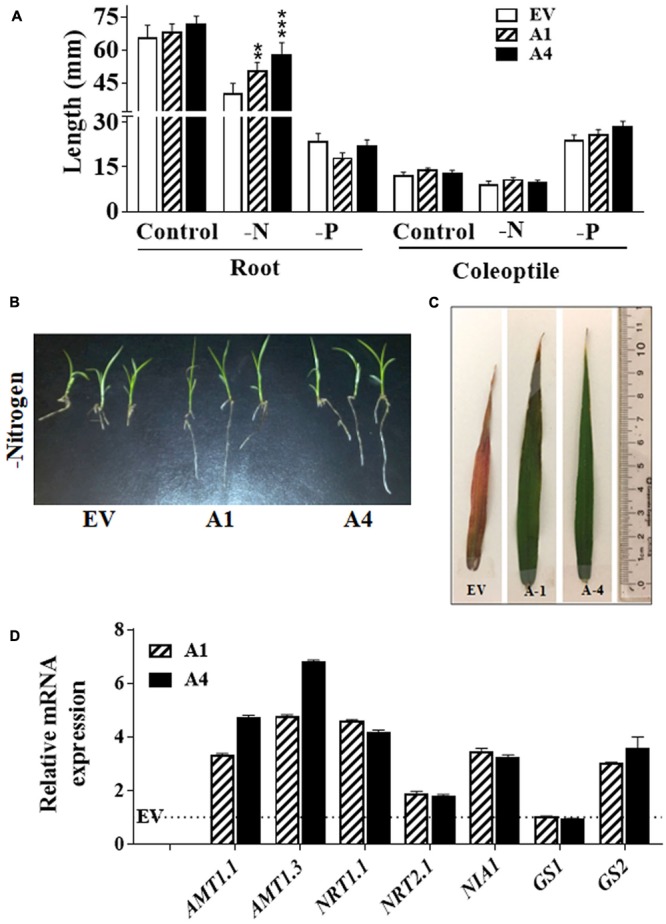
Effect of nitrogen limiting conditions on growth of *AGG3OE* Setaria plants. **(A)** Root and coleoptile lengths of 1 week old seedling grown on 0.5 X MS media lacking nitrogen or phosphate sources. The data represent the mean values (±SE) from sixty seedlings. Asterisks represent *P*-values ≤ 0.01 (^∗∗^) or ≤0.001 (^∗∗∗^) as calculated using Students *t-*test. **(B)** Representative picture of 2 weeks old seedlings of *EV* control and *AGG3OE* lines grown on nitrogen free media. **(C)** Representative picture of the flag leaf of 5 weeks old EV, A1 and A4 plants under nitrogen-limiting condition. **(D)** Expression levels of nitrate transporter and metabolism genes in 2-week old *S. viridis* A1 and A4 plants. Dotted line represents the expression values of the genes (assigned as 1) in EV control plants. The data represent mean values (±SE) of three biological replicates.

Besides stress tolerance, improved nitrogen and phosphate use efficiency of crops is one of the important needs for sustainable agricultural production. Functional study on one of the type III *Gγ* in rice (*DEP1* allele) has shown the regulation of NUE by this protein (Sun et al., 2014). To assess whether *AGG3*-overexpressing transgenic Setaria exhibited improved growth in nitrogen limiting conditions, we compared the primary root and coleoptile length of transgenic lines with EV lines by growing them under nitrogen limiting condition. We observed ∼26% and 44% longer roots in A1 and A4 seedlings, respectively, compared with the EV line; whereas coleoptile lengths were unaffected by the reduction in nitrogen availability (**Figure 3A**). It suggests that the overexpression of *AGG3* in Setaria can improve the root growth at the early seedling stage for maintaining better plant survival in the nitrogen limiting condition. To verify whether the effect of *AGG3*-overexpression was specific to nitrogen we also investigated their early seedling growth under phosphate limiting condition. No differences were seen in root and coleoptile growth between A1, A4 and EV lines (**Figure 3A**) under these conditions, suggesting the role of type III Gγ genes is specific to nitrogen. The transgenic plants continued to exhibit better growth in the nitrogen limiting conditions (**Figure 3B**). When grown in controlled environment growth chambers for 5 weeks under these conditions, the flag leaves of EV containing plants showed clear nitrogen-responsive chlorosis, accumulated anthocyanin and senesced; whereas the flag leaves of A1 and A4 transgenic plants remained green and exhibited no stress-related phenotype (**Figure 3C**). We compared the transcript levels of key transporters and signaling proteins related to nitrogen uptake, sensing and metabolism in EV control versus A1 and A4 transgenic plants (**Figure 3D**). Several of these genes showed modest (2–5 fold) increase in A1 and A4 compared to the EV plants, suggesting that a general improvement in the NUE of these plants is likely correlated with better uptake and/or sensing.

Overall these data suggest that for seed germination and early plant development, the type III Gγ proteins are a direct, positive regulator of stress responses and their role seems to be conserved between dicot and monocot plants, possibly independent of specific variant of the gene present in the genome.

### Effect of *AGG3* Overexpression on Overall Plant Growth, Development and Yield

The type III Gγ proteins are also a major determinant of organ size, especially reproductive organs and seeds. In Arabidopsis, overexpression of *AGG3* results in significantly larger floral organs and bigger seeds whereas opposite was seen with the loss-of-function *agg3* mutant plants (Chakravorty et al., 2011; Li et al., 2012b). Likewise, overexpression of *AGG3* in Camelina increased the seed size and number, in addition to improved biomass production (Roy Choudhury et al., 2014). To determine the effect of *AGG3* overexpression on yield traits reported to be regulated by *DEP1* or *GS3* in rice (Fan et al., 2006; Huang et al., 2009; Mao et al., 2010; Li et al., 2012a), we grew the EV containing and *AGG3* overexpressing plants in greenhouses for their entire life cycle and recorded multiple growth and development traits starting 1 week post-germination for 8 weeks, till the plants were left for drying. Final seed yield was quantified from completely dried plants.

**FIGURE 4:**
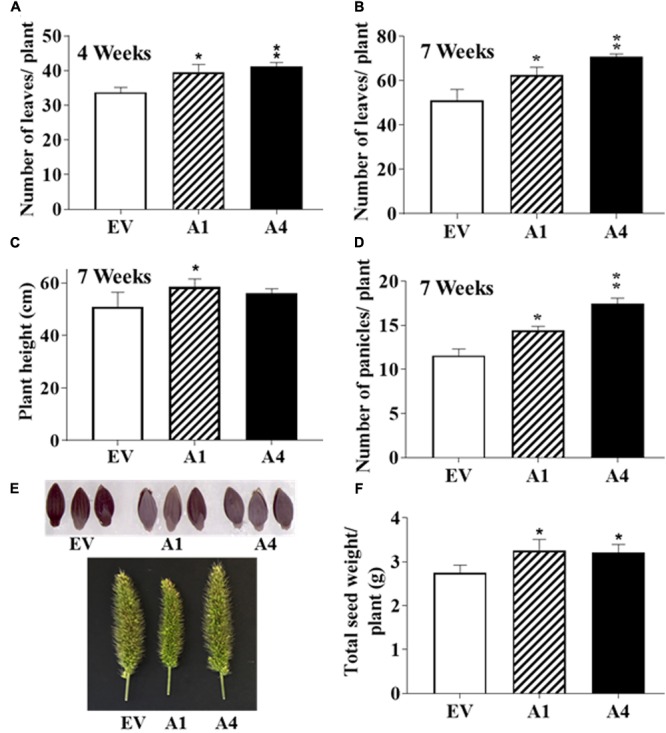
Effect of *AGG3OE* on adult *S. viridis* plants. Adult plant phenotypes of EV, A1 and A4 plants grown under control conditions. Twelve plants per genotype for each of the transgenics were evaluated. The parameters measured were **(A)** number of leaves at 4-weeks, **(B)** leaf number at 7-weeks, **(C)** plant height at 7-weeks, **(D)** number of panicles per plant at 7-weeks. **(E)** Representative pictures of the panicles and seeds of EV versus A1 and A4 transgenic plants. **(F)** The total seed yield per plant. The data represent mean values (±SE) of three biological replicates. Asterisks represent *P*-values ≤ 0.05 (^∗^) or ≤0.01 (^∗∗^) as calculated using Students *t-*test.

After the first 2 weeks when all plants were indistinguishable from each other, the A1 and A4 plants displayed more robust growth. By 4 weeks, the transgenic plants produced a higher number of leaves per plant compared to the EV containing plants (**Figure 4A**). No difference in the flowering time was observed and the first panicle emerged at a similar time for both EV containing and *AGG3* overexpressing plants. At 7 weeks, when the plants had fully matured, the A1 and A4 plants maintained the higher leaf numbers (**Figure 4B** and Supplementary Table S3) and were taller than the EV containing plants (**Figure 4C** and Supplementary Table S3), resulting in an overall increased biomass of the transgenic plants.

The overexpression of *AGG3* resulted in more panicles per plant as recorded at 7 weeks (**Figure 4D** and Supplementary Table S3); however, no difference in the panicle length, density or erectness was observed. The panicles from EV plants were phenotypically indistinguishable from the A1 and A4 plants (**Figure 4E**). Finally, we measured the seed size and seed weight from the A1 and A4 plants and compared it to EV plants. No differences in seed size (**Figure 4E**) or seed weight (100 seed weight per genotype) were observed. However, due to the presence of more panicles per plant, the overall seed produced from A1 and A4 plants was significantly higher than the EV plants (**Figure 4F** and Supplementary Table S3), translating into improved yield per plant.

We also tested the effect of low water stress and nitrogen limiting growth conditions, individually and in combination, on various growth parameters and yield of EV, A1 and A4 plants. Although the transgenic plants exhibited improved stress tolerance at the seed germination and seedling stage and in the growth chambers, in the greenhouse conditions various growth parameters and the yield of A1 and A4 plants were affected by the stress treatment to the similar extent as the EV plants and no noticeable improvement was seen under any of the above listed stress conditions tested (Supplementary Table S3). The *AGG3* transgene was expressed at a significantly higher level in plants grown in the greenhouse, although its expression level was lower under stress conditions compared to the control conditions (Supplementary Figure S4).

These data indicate that while some of the phenotypes ascribed to the regulation by type III Gγ proteins might have a simple causal relationship with the gene expression level; others are more complex and may depend on the presence of specific protein variant or specific environmental conditions.

## Discussion

Naturally occurring or engineered changes in the expression of type III Gγ-proteins result in profound changes in plant architecture, abiotic stress responses and yield potential (Huang et al., 2009; Mao et al., 2010; Botella, 2012; Li et al., 2012a,b; Roy Choudhury et al., 2014; Wendt et al., 2016; Zhao et al., 2016). The distinctive architecture of these proteins, the presence of an extremely Cys-rich region (likely one of the highest Cys containing proteins in nature), and the presence of certain unusual domains (e.g., TNFR or Sprouty) makes them a novel component of the conventional G-protein complex and suggests their possibly unique mechanism of action, which remains largely unknown. The type III Gγ-proteins proteins are clearly a part of the G-protein heterotrimer, as has been confirmed by multiple protein–protein interaction studies as well as genetic analysis (Sun et al., 2014; Wolfenstetter et al., 2014). Homology searches show relatively high sequence conservation in the Gγ domain within the type III family (Supplementary Table S2) as well as when compared with type I or type II family proteins. The C terminal region of the proteins is highly variable, both in its length which could range from 100 to 400 amino acids, as well as in its sequence. However, this region is critical for the proteins’ function, as shown by analysis of rice *GS3* and *DEP1* alleles and by complementation studies in Arabidopsis and rice (Li et al., 2012b; Sun et al., 2014; Wolfenstetter et al., 2014). Most of the naturally occurring mutations that define panicle branching or grain size in rice map to the C-terminal region of DEP1 and GS3, respectively (Mao et al., 2010; Botella, 2012; Wendt et al., 2016). Moreover, the phenotypes of the Arabidopsis *agg3* mutants cannot be complemented with the N-terminal Gγ-like domain and the C-terminal region is required to restore the wild-type phenotypes. Surprisingly, no effect of the deletion of specific domains within the C-terminal region was observed. Mutant *agg3* plants transformed with variants of *AGG3*, which were missing the TNFR, TM or VWFC regions, exhibited WT phenotypes.

In order to determine the role of higher expression levels of a prototypical type III Gγ protein, we chose to overexpress a monocot codon optimized *AGG3* gene in Setaria. Because the overexpression of this gene results in multiple growth and development phenotypes in Arabidopsis and in Camelina (Chakravorty et al., 2011; Li et al., 2012b; Roy Choudhury et al., 2014), it allowed for a direct comparison between phenotypes which are directly correlated with the expression level versus those which are dependent of the presence of specific allelic variants of the gene in the genome.

Our data show both similarities and dissimilarities with the effect of overexpression of *AGG3* in Camelina versus Setaria. Camelina seedlings overexpressing *AGG3* are robust and show a clearly improved growth early on, which is distinguishable from the wild type or EV plants. This was not seen in *Setaria AGG3* overexpressors. One possibility is that the Camelina seeds overexpressing *AGG3* are significantly bigger than the EV seeds resulting in better nutrient availability to the germinating seeds, which is not the case with Setaria *AGG3* overexpressing seeds (**Figure 4E**). However, after 2 weeks an improvement in growth of Setaria *AGG3OE* lines was observed, as seen by more leaves per plant and relatively taller plants compared to the EV plants. An improved growth of plants and more branching was also seen in Camelina *AGG3OE* plants.

The seedling stress responses of Setaria *AGG3-OE* lines were similar to what has been reported for Arabidopsis and Camelina *AGG3-OE* lines. The plants showed less sensitivity to ABA, glucose and NaCl and exhibited improved seedling growth compared to the EV containing seedlings on media containing these additives (**Figure 2**). These responses are predicted to be mediated by the classic G-protein signaling pathways, and therefore, are potentially conserved between different plant species. However, unexpectedly we did not see an effect of improved stress tolerance in mature plants grown under greenhouse conditions. Both *EV* containing and *AGG3-OE* lines of Setaria responded similarly to low water stress. Similar trend was seen in response to nitrogen limiting conditions, where the plants exhibited clearly improved growth at the seedling stage and at the young plant stage (**Figure 3**), but the overall growth, development and yield of mature plants was affected similarly in EV versus *AGG3-OE* lines. Incidentally, rice plants possessing specific *DEP1* alleles have been shown to exhibit significantly improved NUE. The lack of improved stress response of adult plants could be due to the specific growth conditions used in our experiments or due to the fact that *Setaria viridis* is an undomesticated plant and therefore has mechanisms to overcome stresses during the growth over its life cycle. Additionally, a developmental stage dependent effect of AGG3 on plants’ stress tolerance cannot be ruled out.

One of the most crucial phenotypes ascribed to the type III Gγ proteins is the regulation of grain size and panicle density and erectness. These were the traits that led to the discovery and cloning of the rice homologs of these proteins, GS3 and DEP1 (Fan et al., 2006; Huang et al., 2009; Mao et al., 2010; Xu et al., 2016). Even though we observed a clear difference in panicle number per plants in the transgenic lines, which resulted in improved yield (**Figure 4**), the panicle morphology, seed morphology and the seed size of the *AGG3-OE* plants were indistinguishable from the EV control plants. This is surprising as the overexpression of the same gene in Arabidopsis and in Camelina resulted in significantly larger floral organs and seeds. It may be that AGG3 type proteins interact differently with the developmental programs that control seed size in dicots versus monocots. While in dicots, there seems to be a direct positive correlation between the organ size and protein expression level, the regulation seems to be much more complex in monocots and might involve specific regions of the protein or interaction with specific protein complexes.

Overall, our data confirm that at least a subset of the type III Gγ protein regulated processes are directly linked to the gene’s expression level. These include major agronomical traits such as an improved biomass and seed yield. However, our data also emphasize that the roles of these proteins are complex and there are possible allele specific regulatory circuits. The proteins are clearly an important target for breeding or engineering of important traits in plants, and a thorough investigation of different domains, specific regions or specific variants, under different environmental conditions is required, especially in the context of their role as a part of the G-protein heterotrimer, to fully harness their agronomic potential (Botella, 2012). Finally, identification of specific effector proteins, which act downstream of the heterotrimeric G-protein complex, and evaluation of their role in affecting yield and stress-related traits regulated by G-proteins will also be of critical importance. Only few of such effectors are known in plants to date, and some of these may provide for the specificity of response regulation during G-protein signaling.

## Author Contributions

The present study was conceived and directed by SP. JK, SRC, and AV conducted the majority of the experimental work with technical help and contribution from LH, ZR, RP, and DB. SP, JK, SRC, and AV contributed toward designing of experiments, interpretation of results, and writing of the manuscript.

## Conflict of Interest Statement

The authors declare that the research was conducted in the absence of any commercial or financial relationships that could be construed as a potential conflict of interest.
