# Supplementary material for: Arabidopsis Type III Gγ Protein AGG3 Is a Positive Regulator of Yield and Stress Responses in the Model Monocot Setaria viridis
Source: Front Plant Sci. 2018 Feb 9;9:109. doi: 10.3389/fpls.2018.00109 (PMC5811934; doi:10.3389/fpls.2018.00109)
Supplement: Supplementary file 1 [file Table_1.DOCX]

**Table S1. List of primers and probes used in the experiment**

| **Primers used for genotyping transgenic *S. viridis* lines in T_0_ and T_1_ generation** | |
| --- | --- |
| Hpt - F | GAACTCACCGCGACGTCTGTCGAG |
| Hpt - R | AATGACCGCTGTTATGCGGCCAT |
| AGG3 - F | GGCGTCTCTTCTTCTTCTCTG |
| AGG3 - R | CATCAAGGAGGTGTCTGACTTC |
| SiGG3- F | ATATGCGTGCCATGGATCTG |
| SiGG3 - R | GATTGCTCTTGCTGTTCATGC |
| **Primers and probes used for TaqMan assay** | |
| Hpt F3 | ATACGAGGTCGCCAACATCT |
| Hpt R3 | CGTCTGCTGCTCCATACAAG |
| Hpt Probe3 | CCAACCACGGCCTCCAGAAGA |
| SvPCK F3 | CCAACCACGGCCTCCAGAAGA |
| SvPCK R3 | CGTACTTGGTCGGATGGAG |
| SvPCK Probe3 | ACGTTCTCGGCCTGCTTCGG |
| **Primers used for qRT-PCR** | |
| Sv7G171200 F AMT1.1 | CAT CAT GCT CAC CAA CGT GC |
| Sv7G171200 R AMT1.1 | CCG AAG AAC TGC TTC CCG AT |
| SV1G242300 F AMT1.3 | CAT CAT GCT CAC CAA CGT GC |
| SV1G242300 R AMT1.3 | AAG AAG TGC CTG CCG ATG AA |
| SV6G055800 F NRT1.1 | TGT CCG TGT TCT GGC TCA TC |
| SV6G055800 R NRT1.1 | TGC TCA TGG TCT TCA TCC CC |
| Sv1G114800 F NRT2.1 | ACA AGG CCA AGA CCA TCA GG |
| Sv1G114800 R NRT2.1 | GTG GAG ACG AAG CAG GTG AA |
| Sv6G184900 F NIA 1 | ACA AGG CCA AGA CCA TCA GG |
| Sv6G184900 R NIA1 | GTG GAG ACG AAG CAG GTG AA |
| Sv9G359400 F GS1 | CTG CCA GCA GTG AGG AGA AA |
| Sv9G359400 R GS1 | CAT GGC AAA CCC GTT GAC AG |
| Sv2G382100 F GS2 | GAA CAA GGA TGG CTA CCA CG |
| Sv2G382100 R GS2 | CTG CAT CTG TTC TCC GAA CC |
| Sv9G524500 F α1 | TGC GTG ATT GAA AGC ATG GG |
| Sv9G524500 R α1 | GTC TCT TGC AAA ATC CGC CG |
| Sv9G143600 F β1 | GCC TCA CAA GAT GGA AGG CT |
| Sv9G143600 R β1 | CTG GCC ATT GGG TGC AAA AG |
| Sv1G091400 F γ1 | ACA GCG ATG GAA AGC AAA GC |
| Sv1G091400 F γ1 | TGC GCA GAT CTT GTG GAC C |
| Sv9G160600 F γ1a | AGA AGC TCG AGC AAG AAG CG |
| Sv9G160600 R γ1a | TCT GCT TTG CTT TCC ATC GC |
| Sv8G132300 F γ2 | CACGTGGCCTTGGATCTCTC |
| Sv8G132300 R γ2 | CAGCAATCGGGCAAAACACA |
| Sv6G177400 F γ3a | GGC CGA TCC CAT GAT ACC AG |
| Sv6G177400 R γ3a | CGA AAA GCA CGT GCA CAA CT |
| Sv2G229300 F γ3b | CAC GTG CTG CAA CCT GTC AT |
| Sv2G229300 R γ3b | AAC TTG CAG CAT GAG GGG AG |
| Sv9G375000 F γ3c | GCCTCCAGATGCTGCAAAGA |
| Sv9G375000 R γ3c | TGCTGAGACTGCTCATTGCTT |
| Sv2G158600 F RGS | TAT CTC TGG GCA GTT TGG GC |
| Sv2G158600 R RGS | GGC AAC CGG TGC TTA ACA AA |
