# Supplementary material for: Arabidopsis Type III Gγ Protein AGG3 Is a Positive Regulator of Yield and Stress Responses in the Model Monocot Setaria viridis
Source: Front Plant Sci. 2018 Feb 9;9:109. doi: 10.3389/fpls.2018.00109 (PMC5811934; doi:10.3389/fpls.2018.00109)
Supplement: Supplementary file 2 [file Table_2.DOCX]

**Table S2. Amino acid sequence similarity between *Arabidopsis thaliana* (At), *Oryza sativa* (Os) and *Setaria viridis* (Sv) type III Gγ proteins**

**Amino acid sequence identity (%) of full length type III Gγ proteins.**

|  | AtAGG3 | OsDEP1 | OsGS3 | OsGGC2 | Sevir.GG3a | Sevir.GG3b | Sevir.GG3c |
| --- | --- | --- | --- | --- | --- | --- | --- |
| AtAGG3 | *** | 40.23 | 12.35 | 19.12 | 25.19 | 31.47 | 29.48 |
| OsDEP1 |  | *** | 7.51 | 20.18 | 23.70 | 56.80 | 21.59 |
| OsGS3 |  |  | *** | 15.51 | 20.68 | 20.25 | 45.68 |
| OsGGC2 |  |  |  | *** | 31.04 | 30.74 | 14.62 |
| Sevir.GG3a |  |  |  |  | *** | 54.57 | 24.17 |
| Sevir.GG3b |  |  |  |  |  | *** | 11.39 |
| Sevir.GG3c |  |  |  |  |  |  | *** |

**Amino acid sequence identity (%) of Gγ like domain of the type III Gγ proteins.**

|  | AtAGG3 | OsDEP1 | OsGS3 | OsGGC2 | Sevir.GG3a | Sevir.GG3b | Sevir.GG3c |
| --- | --- | --- | --- | --- | --- | --- | --- |
| AtAGG3 | *** | 40.00 | 22.96 | 31.85 | 29.62 | 35.55 | 34.81 |
| OsDEP1 |  | *** | 28.07 | 64.03 | 50.87 | 71.92 | 35.96 |
| OsGS3 |  |  | *** | 28.03 | 30.84 | 39.25 | 69.15 |
| OsGGC2 |  |  |  | *** | 57.54 | 70.75 | 30.18 |
| Sevir.GG3a |  |  |  |  | *** | 63.80 | 23.14 |
| Sevir.GG3b |  |  |  |  |  | *** | 35.13 |
| Sevir.GG3c |  |  |  |  |  |  | *** |

**Amino acid sequence identity (%) of C-terminal domain of the type III Gγ proteins.**

|  | AtAGG3 | OsDEP1 | OsGS3 | OsGGC2 | Sevir.GG3a | Sevir.GG3b | Sevir.GG3c |
| --- | --- | --- | --- | --- | --- | --- | --- |
| AtAGG3 | *** | 40.51 | 0.00 | 4.31 | 20.68 | 26.72 | 23.27 |
| OsDEP1 |  | *** | 0.00 | 4.16 | 13.78 | 51.28 | 13.14 |
| OsGS3 |  |  | *** | 4.80 | 12.00 | 4.00 | 25.60 |
| OsGGC2 |  |  |  | *** | 17.99 | 11.71 | 7.11 |
| Sevir.GG3a |  |  |  |  | *** | 48.80 | 25.71 |
| Sevir.GG3b |  |  |  |  |  | *** | 5.45 |
| Sevir.GG3c |  |  |  |  |  |  | *** |
