# Supplementary material for: Arabidopsis Type III Gγ Protein AGG3 Is a Positive Regulator of Yield and Stress Responses in the Model Monocot Setaria viridis
Source: Front Plant Sci. 2018 Feb 9;9:109. doi: 10.3389/fpls.2018.00109 (PMC5811934; doi:10.3389/fpls.2018.00109)
Supplement: Supplementary file 3 [file Table_3.DOCX]

|  | **Control** | **WW + low N_2_** | **LW + N_2_** | **LW + low N_2_** |
| --- | --- | --- | --- | --- |
| **Leaf number per plant at 7 weeks** | | | | |
| **EV** | **51.00±7.07** | **28.33±0.68** | **38.83±3.87** | **19.66±1.79** |
| **A1** | **62.55±3.57*** | **32.69±3.08** | **41.88±1.12** | **20.77±0.80** |
| **A4** | **70.66±1.34*** | **32.00±1.52** | **44.08±4.78** | **25.47±1.09*** |
| **Plant height (cm) at 7 weeks** | | | | |
| **EV** | **50.98±5.50** | **44.59±3.48** | **35.16±0.54** | **42.85±6.90** |
| **A1** | **58.47±3.07*** | **56.16±0.58*** | **36.46±1.38** | **56.30±1.71** |
| **A4** | **56.12±1.70*** | **52.47±1.69** | **32.61±2.79** | **46.00±4.56** |
| **Panicle number per plant at 7 weeks** | | | | |
| **EV** | **11.58±1.73** | **4.41±0.83** | **11.16±0.22** | **5.19±0.36** |
| **A1** | **14.44±0.44*** | **5.33±0.38** | **08.94±0.52** | **4.66±0.33** |
| **A4** | **17.44±0.62*** | **5.38±0.20** | **12.33±0.93** | **4.50±0.28** |
| **Terminal panicle length (cm) at 7 weeks** | | | | |
| **EV** | **5.59±0.09** | **5.84±0.24** | **5.79±0.19** | **5.81±0.22** |
| **A1** | **5.62±0.20** | **5.76±0.05** | **5.60±0.25** | **5.23±0.34** |
| **A4** | **5.93±0.15** | **5.75±0.33** | **5.96±0.16** | **5.63±0.21** |
| **Seed weight (gm) per plant** | | | | |
| **EV** | **2.74±0.38** | **1.34±0.14** | **2.05±0.21** | **1.31±0.18** |
| **A1** | **3.50±0.22*** | **1.51±0.14** | **2.06±0.17** | **1.24±0.27** |
| **A4** | **3.40±0.33*** | **1.42±0.05** | **2.01±0.10** | **1.30±0.16** |

**Table S3:** Effect of water and nitrogen deficit on vegetative and reproductive growth parameters of transgenic Setaria plants compared to EV plants. Control- well-watered + 15 mM nitrogen supplemented in the media; WW + low N_2_- well-watered, no nitrogen supplement; LW + N2 - low water (50% capacity compared to the well-watered control) + 15 mM nitrogen, and LW + low N_2_ - low-water and no nitrogen supplement in the media. The data represent the mean values (± SE) from 36 seedlings. Asterisks represent *P*-values ≤ 0.05 as calculated using Students *t* test.
