# Supplementary material for: Arabidopsis Type III Gγ Protein AGG3 Is a Positive Regulator of Yield and Stress Responses in the Model Monocot Setaria viridis
Source: Front Plant Sci. 2018 Feb 9;9:109. doi: 10.3389/fpls.2018.00109 (PMC5811934; doi:10.3389/fpls.2018.00109)
Supplement: Supplementary file 4 [file Image_1.PDF]

>At5g20635(AGG3)
ATGTCTGCTCCTTCTGGCGGTGGCGAAGGAGGAGGAAAAGAATCAGCTGCTGGTGGAGTGAGTTCATCGTCTCTTGC
TCCGTCGTCTCTACCACCGCCTCGTCCTAAGTCTCCACCAGAGTATCCAGATTTGTACGGGAAACGCAGAGAGGCGG
CGAGAGTTCAGATGCTCGAGAGAGAGATTGGTTTTCTCGAGGGCGAAATTAAATTCATCGAAGGCGTACAACCGGCA
TCTAGATGCATCAAAGAAGTCTCTGATTTTGTTGTTGCAAATTCTGACCCATTGATCCCTGCACAACGAAAAAGTCG
AAGATCCTTCCGGTTCTGGAAGTGGCTCTGTGGCCCATGTTTGAGCCTGGTGAGTTTCTGCTGTTGCTGCCAATCCA
AATGTTCGTGCCATCTGAGGAAACCCAAGTGCTGCAACTGTACATCTTGCAGCTGTATAGGGTCCAAATGCTGTGAC
GGGTCATGCTGCTCAAACATTTGTTGTTGCCCGAGACTAAGCTGCCCGAGCTGTTCATGCTTCCGAGGTTGCTGGTG
TTCTTGTCCGGACATGTCTTGCTGCATTCCCAGCTGTTTCCGCAGTTGCAGTTGCACTCGACCGTCGTGTCTGAATA
AAAAGAAGAGCTCATGCTGCAGCTGCAACTGCAAGATCAGATGGTCATCTTGTTTTAGTTGTCCCAAGGTACGACTT
TGTTCTTGTTGTTTTTGCAATTGTAAAAATCTATGTTCTAATCCTTGTTGTTTAGCTTTCTAA

>monocot-codon-optimized_AGG3
ATGTCTGCTCCAAGCGGCGGCGGCGAGGGCGGCGGCAAGGAGTCTGCTGCTGGCGGCGTCTCTTCTTCTTCTCTGGC
TCCAAGCTCTCTCCCACCACCAAGGCCAAAGTCCCCTCCAGAGTACCCAGACCTGTACGGCAAGCGCAGGGAGGCTG
CTCGCGTCCAGATGCTGGAGAGGGAGATCGGCTTCCTGGAGGGCGAGATCAAGTTCATCGAGGGCGTTCAGCCCGCC
TCCAGGTGCATCAAGGAGGTGTCTGACTTCGTGGTCGCCAACAGCGATCCGCTCATCCCAGCTCAGCGCAAGTCTCG
CAGGAGCTTCAGGTTCTGGAAGTGGCTGTGCGGCCCATGCCTCTCCCTGGTTTCTTTCTGCTGCTGCTGCCAGAGCA
AGTGCTCCTGCCACCTCCGCAAGCCAAAGTGCTGCAACTGCACCTCTTGCAGCTGCATCGGCTCCAAGTGCTGCGAC
GGCTCTTGCTGCAGCAACATCTGCTGCTGCCCTAGGCTGTCCTGCCCATCCTGCTCTTGCTTCAGGGGCTGCTGGTG
CTCCTGCCCTGATATGTCTTGCTGCATCCCCAGCTGCTTCCGCAGCTGCTCCTGCACTAGGCCCTCCTGCCTCAACA
AGAAGAAGTCCTCTTGCTGCTCTTGCAACTGCAAGATCCGCTGGAGCTCCTGCTTCTCCTGCCCGAAGGTGAGGCTG
TGCTCCTGCTGCTTCTGCAACTGCAAGAACCTGTGCTCCAACCCGTGCTGCCTCGCTTTCTGATGA

>At5g20635(AGG3)
MSAPSGGGEGGGKESAAGGVSSSSLAPSSLPPPRPKSPPEYPDLYGKRREAARVQMLERE
IGFLEGEIKFIEGVQPASRCIKEVSDFVVANSDPLIPAQRKSRRSFRFWKWLCGPCLSLV
SFCCCCQSKCSCHLRKPKCCNCTSCSCIGSKCCDGSCCSNICCCPRLSCPSCSCFRGCWC
SCPDMSCCIPSCFRSCSCTRPSCLNKKKSSCCSCNCKIRWSSCFSCPKVRLCSCCFCNCK
NLCSNPCCLAF*

>monocot-codon-optimized_AGG3
MSAPSGGGEGGGKESAAGGVSSSSLAPSSLPPPRPKSPPEYPDLYGKRREAARVQMLERE
IGFLEGEIKFIEGVQPASRCIKEVSDFVVANSDPLIPAQRKSRRSFRFWKWLCGPCLSLV
SFCCCCQSKCSCHLRKPKCCNCTSCSCIGSKCCDGSCCSNICCCPRLSCPSCSCFRGCWC
SCPDMSCCIPSCFRSCSCTRPSCLNKKKSSCCSCNCKIRWSSCFSCPKVRLCSCCFCNCK
NLCSNPCCLAF**

Figure S1. Chemically synthesized Arabidopsis *AGG3* (*At5g20635*) gene sequence by codon optimization using the monocot-preferred codons.
