# Supplementary material for: Arabidopsis Type III Gγ Protein AGG3 Is a Positive Regulator of Yield and Stress Responses in the Model Monocot Setaria viridis
Source: Front Plant Sci. 2018 Feb 9;9:109. doi: 10.3389/fpls.2018.00109 (PMC5811934; doi:10.3389/fpls.2018.00109)
Supplement: Supplementary file 5 [file Image_2.PDF]

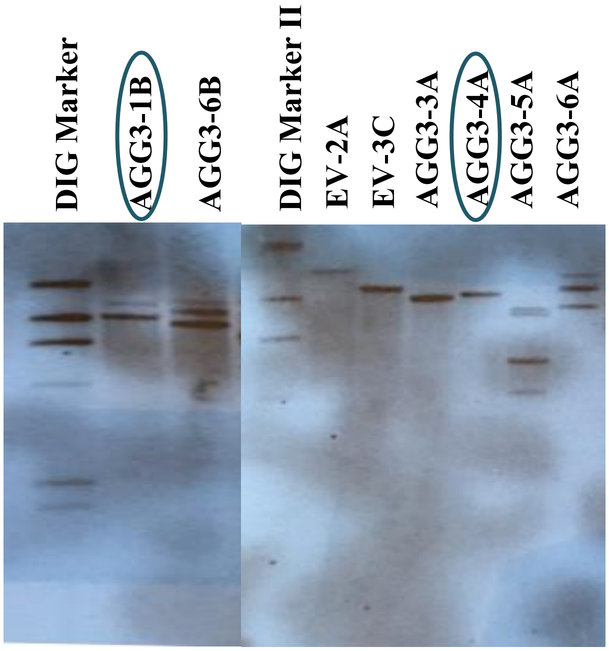

Figure S2. Southern blot analysis of transgenic *S. viridis* lines. *MfeI* digested genomic DNA from various lines was hybridized with a probe complementary to *Hyg* gene. EV-2A, -3C, AGG3-3A, -4A are single copy; AGG3-1B are two copy and AGG3-5A, -6A, -1B and -6B are three copy events.
