# Supplementary material for: Arabidopsis Type III Gγ Protein AGG3 Is a Positive Regulator of Yield and Stress Responses in the Model Monocot Setaria viridis
Source: Front Plant Sci. 2018 Feb 9;9:109. doi: 10.3389/fpls.2018.00109 (PMC5811934; doi:10.3389/fpls.2018.00109)
Supplement: Supplementary file 6 [file Image_3.PDF]

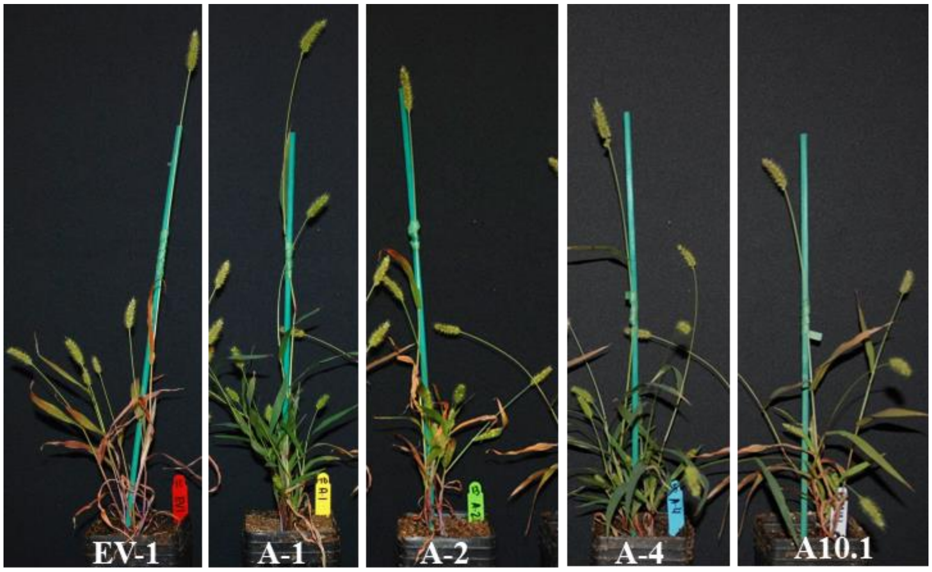

Figure S3: Phenotype of adult Setaria plants overexpressing *AGG3* transgene in multiple T3 lines. Plants were grown in growth chambers for 1 month. EV; plants with empty vector control, A1, A2 and A4; lines with independent transgenic events. A10.1 is the wild type Setaria accession used for transformation.
