# Supplementary material for: Arabidopsis Type III Gγ Protein AGG3 Is a Positive Regulator of Yield and Stress Responses in the Model Monocot Setaria viridis
Source: Front Plant Sci. 2018 Feb 9;9:109. doi: 10.3389/fpls.2018.00109 (PMC5811934; doi:10.3389/fpls.2018.00109)
Supplement: Supplementary file 7 [file Image_4.PDF]

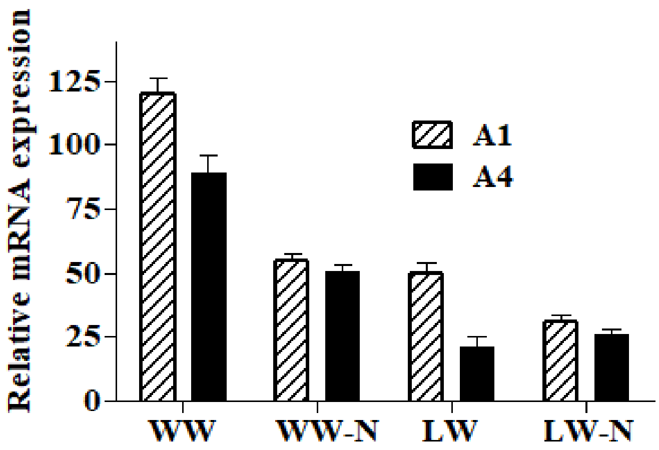

Figure S4: *AGG3* transgene expression analysis in green house grown, stress-treated adult plants. Transcript level of *AGG3* gene was determined in the one month old adult Setaria plants grown in the green-house condition under different treatments using real time qRT-PCR. The expression values of EV, A1 and A4 were normalized with ubiquitin gene and the fold change was determined as compared to the EV control (Assigned as 1). The values are presented as the mean ± SEM of three biological replicates. EV, Setaria plants overexpressing the empty vector; A1, A4 are the two independent transgenic lines. WW, WW-N, LW, LW-N are the different treatment conditions.
